# Identification of *Acinetobacter baumannii* and its carbapenem-resistant gene *bla*_*OXA-23-like*_ by multiple cross displacement amplification combined with lateral flow biosensor

**DOI:** 10.1038/s41598-019-54465-8

**Published:** 2019-11-29

**Authors:** Shoukui Hu, Lina Niu, Fan Zhao, Linlin Yan, Jinqing Nong, Chunmei Wang, Naishu Gao, Xiaoxue Zhu, Lei Wu, Tianhui Bo, Hongyu Wang, Jin Gu

**Affiliations:** 1 Department of Clinical Laboratory, Peking University Shougang Hospital, Beijing, 100144 China; 2 Department of Pathogen Biology, School of Basic Medicine and Life science, Hainan Medical University, Key Laboratory of Translation Medicine Tropical Diseases, Hainan Medical University-University of Hong Kong Joint Laboratory of Tropical Infectious Diseases, Haikou, 571101 China

**Keywords:** Applied microbiology, Clinical microbiology

## Abstract

*Acinetobacter baumannii* is a frequent cause of the nosocomial infections. Herein, a novel isothermal amplification technique, multiple cross displacement amplification (MCDA) is employed for detecting all *A. baumannii* strains and identifying the strains harboring *bla*_*OXA-23-like*_ gene. The duplex MCDA assay, which targets the *pgaD* and *bla*_*OXA-23-like*_ genes, could identify the *A. baumannii* isolates and differentiate these isolates harboring *bla*_*OXA-23-like*_ gene. The disposable lateral flow biosensors (LFB) were used for analyzing the MCDA products. A total of sixty-eight isolates, include fifty-three *A. baumannii* strains and fifteen non-*A. baumannii* strains, were employed to optimize MCDA methods and determine the sensitivity, specificity and feasibility. The optimal reaction condition is found to be 63 °C within 1 h, with limit of detection at 100 fg templates per tube for *pgaD* and *bla*_*OXA-23-like*_ genes in pure cultures. The specificity of this assay is 100%. Moreover, the practical application of the duplex MCDA-LFB assay was evaluated using clinical samples, and the results obtained from duplex MCDA-LFB method were consistent with conventional culture-based technique. In sum, the duplex MCDA-LFB assay appears to be a reliable, rapid and specific technique to detect all *A. baumannii* strains and identify these strains harboring *bla*_*OXA-23-like*_ gene for appropriate antibiotic therapy.

## Introduction

*Acinetobacter. spp* is a genus of gram-negative, strictly aerobic, non-fermenting, oxidase negative coccobacillus^1^. It is a diverse genus, several species of which belong to the normal flora on the skin and mucosa of human beings. *Acinetobacter baumannii* (*A. baumannii*), as one pathogenic species of *Acinetobacter spp*., is a frequent cause of the nosocomial infections, which manifest as pneumonia, bloodstream infection, skin or soft tissue infections, urinary tract infection and meningitis^2^. Numerous outbreaks caused by *A. baumannii* had been reported, which mainly occurred in the intensive care units (ICUs)^2,3^. With the widely occurrence of multi-drug resistance, *A. baumannii* is an increasing menace for the hospitalized patients, especially for the severely immuno-compromised ones in the ICUs. Besides, some *A. baumannii* strains are capable of forming biofilms and show tolerance to desiccation, which further contribute to their maintenance in the hospitals^4,5^.

Carbapenem antibiotics are the suggested candidates for the treatment of infections caused by the multidrug-resistant bacteria^6^. However, resistance to carbapenem is increasingly becoming a great concern, especially among the nosocomial strains belonging to the ESKAPE group of pathogens (i.e. *Enterococcus faecium*, *Staphylococcus aureus*, *Klebsiella pneumoniae*, *A. baumannii*, *Pseudomonas aeruginosa* and *Enterobacter species*)^7^. Carbapenem resistance of *A. baumannii* is mainly mediated by the *bla*_*OXA*_ genes (like the *ISAbal*-*bla*_*OXA-51-like*_, *bla*_*OXA-58-like*_, and *bla*_*OXA-40-like*_ genes), which encode the carbapenem-hydrolyzing class D β-lactamases^1^. The *bla*_*OXA-23-like*_ gene is one of the most prevalent β-lactamase genes on the genome (mostly on the plasmids) of carbapenem-resistant *A. baumannii*^1,8^. Specific and rapid identification of *A. baumannii* and the strains harboring *bla*_*OXA-23-like*_ gene, will offer referential information on the therapeutic and control precautions for the nosocomial infections owing to the carbapenem-resistant *A. baumannii*.

Generally, reliable identification of target pathogen has been primarily relying on culture-based technique that often fails to provide valuable results in time^9^. Although molecular-based assays (such as PCR-based assays) are more sensitive and rapid than culture-based methods, these results are often not in conformity with culture-based methods and challenged by doubts on false-positive results^10^. Moreover, the nucleic acids-based methodologies often rely on expensive laboratory apparatus^11^. As progress is made towards better infection control, more rapid, simple and sensitive methods will be needed for reliably detect target *A. baumannii* and accurately identify carbapenem-resistant *A. baumannii*. Thus, further efforts are required for establishing next generation diagnostic technologies for the use in field, “on-site” and clinical settings, and replacing the existing diagnostic tools for target pathogen detection.

The recently established multiple cross displacement amplification (MCDA) assay was a powerful innovative nucleic acid amplification technique^12^. MCDA was based on strand displacement nucleic acid synthesis in the presence of *Bst* polymerase under isothermal conditions. A total of ten primers were employed to recognize ten distinct regions on the target gene. Given that this technique eliminated the use of a thermocycler, and did not require sophisticated training, thus MCDA showed the potential as a valuable diagnostic tool for field testing and point-of-care diagnosis^13^. Similar to other isothermal amplification methods like LAMP (loop-mediated isothermal amplification) and CPA (cross-priming amplification), the amplification products were a combination of different sequences with varying fragment sizes, which introduce an obstacle for the multiplex amplification^14^. The label-based lateral flow biosensor (LFB) makes it possible for the multiple identifications by detecting the amplification products labeled with different biomarkers^3,14^. The portable and dry-proof gold nanoparticle LFB is simple and reliable technique showing the amplification results within a few minutes of reaction. Thus, the LFB scheme was introduced in this study to identify the genus *A. baumannii* and its prevalent carbapenem resistant gene *bla*_*OXA-23-like*_ at the same time. However, it was found that the classic double labeled primers for the LFB testing may introduce a false positive result owing to the hybridization between the labeled primers^14^. Recently, Wang *et al*. reported a new LFB technique of single labeling which can generate the double labeled amplicons as well and successfully eliminated the hybridization interaction of labeled primers^14,15^.

The aim of the study is to develop a rapid and simple duplex-MCDA-LFB assay for the detection of *A. baumannii* and differentiation of the strains harboring the prevalent carbapenem resistant gene *bla*_*OXA-23-like*_. The single labeling technique was introduced to avoid the hybridization between labeled primers. And sputum samples from clinical patients were used for the applicability evaluation.

## Results

### Validation and confirmation of *pgaD*- and *bla*_*OXA-23-like*_-MCDA products

**Figure 1:**
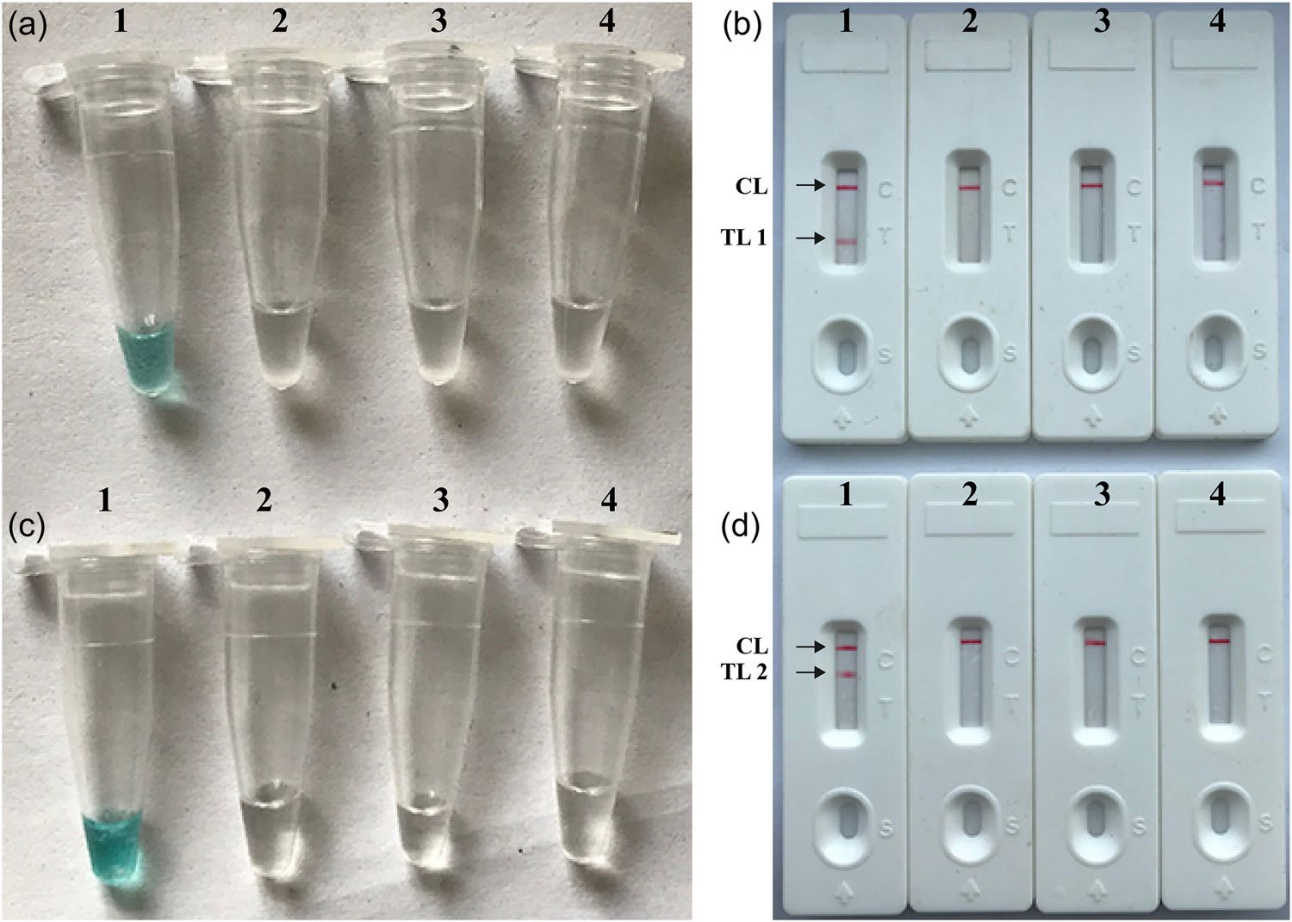
Detection and confirmation of *pgaD*- and *bla*_*OXA-23-like*_-MCDA products. (**a,c**), Color change of *pgaD*- and *bla*_*OXA-23-like*_ -MCDA tubes; (**b**,**d**), LFB applied for visual detection of *pgaD*- and *bla*_*OXA-23-like*_-MCDA products. Tube a1 (biosensor b1), positive amplification; tube a2 (biosensor b2), negative amplification (*K. pneumoniae*); tube a3 (biosensor b3), negative amplification (*S. aureus*); tube a4 (biosensor b4), negative control (DW); Tube c1 (biosensor d1), positive amplification; tube c2 (biosensor d2), negative amplification (*K. pneumoniae*); tube c3 (biosensor d3), negative amplification (*S. aureus*); tube c4 (biosensor d4), negative control (DW).

To demonstrate the feasibility of the *pgaD*- and *bla*_*OXA-23-like*_-MCDA primers, *pgaD*- and *bla*_*OXA-23-like*_-MCDA amplifications were conducted in the absence and presence of target DNAs for 60-min under the isothermal conditions (63 °C). As shown in Fig. 1, the positive *pgaD*- and *bla*_*OXA-23-like*_-MCDA tubes could be visualized with the unaided eye as being lake green, and the amplification vessels containing the blank control and negative control remained colorless (Fig. 1a,c). By lateral flow biosensor, two red bands (TL1 and CL) were observed in the positive *pgaD*-MCDA amplification (Fig. 1b), and two red lines (TL2 and CL) appeared on the lateral flow biosensor in the positive *bla*_*OXA-23-like*_-MCDA amplification (Fig. 1d). Particularly, only a red line (CL) was observed in the blank and negative controls (Fig. 1b,d). Together, our data verified that the *pgaD*- and *bla*_*OXA-23-like*_-MCDA primer sets were valuable candidates for development of the *pgaD*-MCDA-LFB,* bla*_*OXA-23-like*_-MCDA-LFB and duplex MCDA-LFB assays for target gene detection.

### Screening of optimal amplification temperature

**Figure 2:**
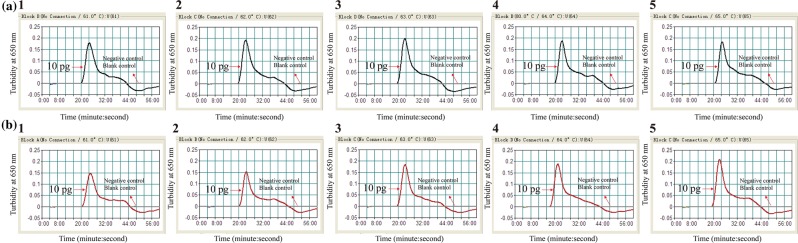
Optimal amplification temperature for *pgaD*- and *bla*_*OXA-23-like*_-MCDA primer sets. The MCDA amplifications for detection of *pgaD* (**a**) and *bla*_*OXA-23-like*_ (**b**) were monitored by real-time measurement of turbidity and the corresponding curves of concentrations of templates were marked in the figures. The threshold value was 0.1 and the turbidity of >0.1 was considered as positive. Five kinetic graphs (1–5) were generated at various temperatures (61 °C-65 °C, 1 °C intervals) with target pathogens DNA. (**a**) graphs from 2 (62 °C) to 4 (64 °C) showed robust amplification; (**b**) graphs from 2 (62 °C) to 4 (64 °C) showed robust amplification.

Then, we screened the optimal amplification temperatures of *pgaD*- and *bla*_*OXA-23-like*_- MCDA primer sets by observing the real time turbidity changes at different temperatures (61 °C to 65 °C with 1 °C interval) for 60 min. DNA templates of SG-AB001 at the level of 10 pg per tube were used for the optimal amplification temperature screening. As shown in Fig. 2, the optimal amplification temperatures for both *pgaD*- and *bla*_*OXA-23-like*_-MCDA primer sets were within the range of 62 °C to 64 °C. A amplification temperature of 63 °C was used in the following study.

### Sensitivity of the single and duplex MCDA-LFB assay

**Figure 3:**
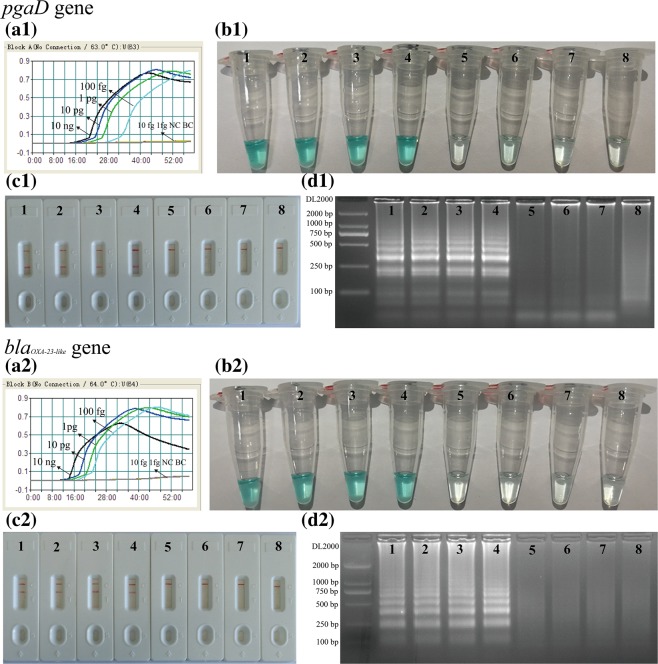
Detection of a single target in a MCDA reaction. Two sets of MCDA primers targeting the *pgaD* (**a1**,**b1**,**c1**,**d1**) and *bla*_*OXA-23-like*_ (**a2**,**b2**,**c2**,**d2**) genes were used in different reactions and the serial dilutions (10 ng, 10 pg, 1 pg, 100 fg, 10 fg and 1 fg) of target templates were subjected to MCDA reactions. (**a1**) and (**a2**), real-time turbidity applied for analysis of *pgaD*- and *bla*_*OXA-23-like*_-MCDA products. (**b1**) and (**b2**), MG applied for analysis of *pgaD*- and *bla*_*OXA-23-like*_-MCDA products. (**c1**) and (**c2**), LFB applied for analysis of *pgaD*- and *bla*_*OXA-23-like*_-MCDA products. (**d1**) and (**d2**), gel electrophoresis applied for analysis of *pgaD*- and *bla*_*OXA-23-like*_-MCDA products. Signals/Tubes/Biosensors/Lanes 1–6: *A. baumannii* (SG-AB001) genomic templates (10 ng-1fg); Signal/Tube/Biosensor/Lane 7: negative control (*K. pneumoniae*); Signal/Tube/Biosensor/Lane 8: blank control (DW). The d1 and d2 were cropped from different gels, the full-length gels can be found as Supplementary Fig. S1.

The limit of detection (LoD) of *pgaD*- and *bla*_*OXA-23-like*_-MCDA assays was firstly evaluated by examining the diluted DNA templates of SG-AB001 (10 ng/μL, 10 pg/μL, 1 pg/μL, 100 fg/μL, 10 fg/μL and 1 fg/μL). The reaction mixtures with DNA template of *K. pneumoniae* (ATCC2146) were used as negative control, and DW for blank control. The LoD for *pgaD*-MCDA assay was 100 fg (Fig. 3-a1, b1,c1, –d1). For positive amplifications of *pgaD*-MCDA assay, there were two red bands (TL1 and CL) appeared on LFB (Fig. 3-c1). Analytical sensitivity of *bla*_*OXA-23-like*_-MCDA assay was also 100 fg (Fig. 3-a2,b2,c2,d2), and two red bands (TL2 and CL) were seen in positive amplifications of *bla*_*OXA-23-like*_-MCDA reactions (Fig. 3-c2). By LFB, only a red line was visible in the negative and blank control for *pgaD*- and *bla*_*OXA-23-like*_-MCDA assays (Fig. 3-c1,c2). Analysis of *pgaD*- and *bla*_*OXA-23-like*_-MCDA results using LFB was in complete accordance with these monitoring techniques (real-time turbidity, color indicator and gel electrophoresis detection) employed in this report (Fig. 3).

**Figure 4:**
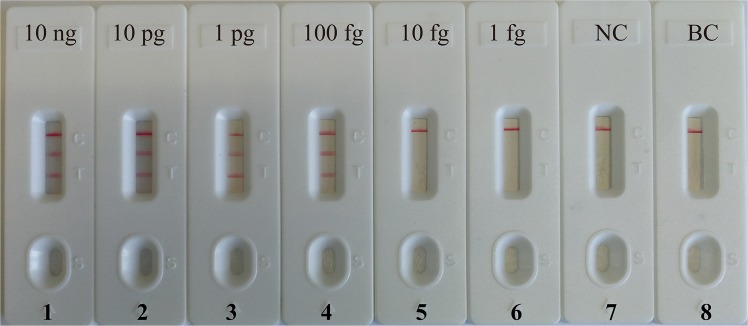
Detection of multiplex targets in a m-MCDA reaction. Two sets of MCDA primers targeting *pgaD*- and *bla*_*OXA-23-like*_-MCDA genes were simultaneously added to a reaction tube and the LoD of m-MCDA for simultaneously detecting *pgaD* and *bla*_*OXA-23-like*_ genes was confirmed using LFB. Biosensors 1, 2, 3, 4, 5 and 6 represent DNA levels of 10 ng (*A. baumannii* SG-AB001), 10 pg (*A. baumannii* SG-AB001), 1 pg (*A. baumannii* SG-AB001), 100 fg (*A. baumannii* SG-AB001), 10 fg (*A. baumannii* SG-AB001) and 1 fg (*A. baumannii* SG-AB001); biosensor 7, negative control (*K. pneumoniae*); biosensor 8, blank control (DW). The LoD of m-MCDA assay for *pgaD* and *bla*_*OXA-23-like*_ detection was 100 fg per vessel.

Following this, we examined the detection limit of the duplex MCDA reactions, which can simultaneously detect *pgaD* and *bla*_*OXA-23-like*_ genes. The amplicons generated from duplex MCDA reactions were directly analyzed using the biosensor. As shown in Fig. 4, three red lines, including TL1, TL2 and CL, appeared on the LFB, indicating positive reactions for *pgaD* and *bla*_*OXA-23-like*_ detection. However, only a red band (CL) appeared on the biosensor, reporting negative reactions at the concentration lower than 10 fg of templates per reaction, negative control and blank control. Analytical sensitivity of the duplexed MCDA-LFB method for simultaneous detecting *pgaD* gene and *bla*_*OXA-23-like*_ gene was also 100 fg per reaction, which was consistent with the LoD of the single *pgaD-* and *bla*_*OXA-23-like*_-MCDA-LFB methods (Figs. 3 and 4).

### Specificity of the MCDA-LFB assay

**Figure 5:**
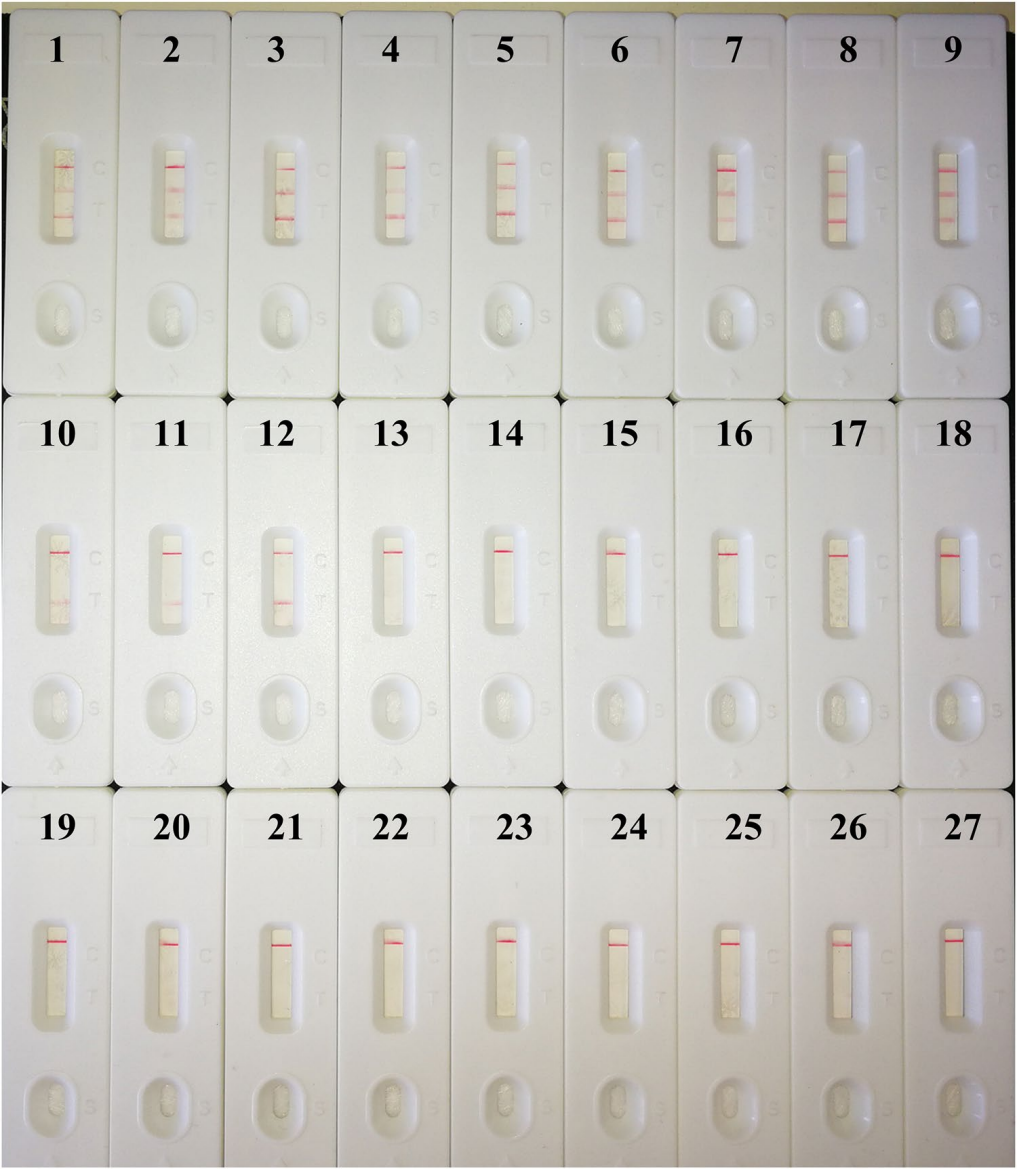
Specificity of duplex MCDA-LFB assay using different bacterial strains. The m-MCDA amplifications were carried out using different genomic DNA templates and the results were indicated using LFB. Biosensors 1–9, *A. baumannii* strains with *bla*_*OXA-23-like*_ gene; Biosensors 10–12, *A. baumannii* strains without *bla*_*OXA-23-like*_ gene; biosensors 13–27, *Listeria monocytogenes*, *Bacillus cereus*, *Citrobacter braakii*, *Citrobacter freundii*, *Corynebacterium ammoniagenes*, *Escherichia coli*, *Klebsiella pneumoniae*, *Proteus mirabilis*, *Providencia rettgeri*, *Pseudomonas aeruginosa*, *Serratia marcescens*, *Serratia marcescens*, *Staphylococcus aureus*, *Staphylococcus epidermidis*, *Streptococcus salivarius*.

Analytical specificity of duplex MCDA-LFB method is examined using extracted genomic DNA from 53 *A. baumannii* strains and 15 non-*A. baumannii* strains. All positive results were obtained only with the templates extracted from *A. baumannii* strains (Fig. 5 and Table 1). Three red bands (TL1, TL2 and CL) appeared on the biosensor, indicating the positive results for *A. baumannii* strains with the carbapenem resistance related gene *bla*_*OXA-23-like*_. TL1 and CL lines appeared on the biosensor, indicating the positive results for *A. baumannii* strains without the carbapenem resistance related gene *bla*_*OXA-23-like*_. Only CL line appeared on the biosensor, indicating the negative results for non-*A. baumannii* strains and negative control. The duplex MCDA-LFB assay could simultaneously detect and correctly identify *pgaD* and *bla*_*OXA-23-like*_ genes in a single reaction system. No cross-reactions to non-*A. baumannii* isolates were obtained according the specificity analysis, thus the specificity of this assay was of 100% (Table 1). These data demonstrate that the duplex MCDA-LFB assay has high selectivity of *A. baumannii* strains.

**Table 1:** Bacterial strains used in the study.

Strain ID	Strains	Sources of strains (No. of strains)^a^	Duplex MCDA-LFB ^b^	
			*pgaD*	*bla*_*OXA-23-like*_
1	*Acinetobacter baumannii*	SG-AB001	P	P
2–50	*Acinetobacter baumannii*	Isolated strain(49)	P	P
51–53	*Acinetobacter baumannii*	Isolated strains (3)	P	N
54	*Listeria monocytogenes*	Isolated strains (1)	N	N
55	*Bacillus cereus*	Isolated strain (1)	N	N
56	*Citrobacter braakii*	Isolated strain (1)	N	N
57	*Citrobacter freundii*	Isolated strain (1)	N	N
58	*Corynebacterium ammoniagenes*	Isolated strain (1)	N	N
59	*Escherichia coli*	ATCC35218	N	N
60	*Klebsiella pneumoniae*	ATCC2146	N	N
61	*Proteus mirabilis*	Isolated strain (1)	N	N
62	*Providencia rettgeri*	Isolated strain (1)	N	N
63	*Pseudomonas aeruginosa*	ATCC27853	N	N
64	*Serratia marcescens*	Isolated strain (1)	N	N
65	*Serratia marcescens*	Isolated strain (1)	N	N
66	*Staphylococcus aureus*	ATCC29213	N	N
67	*Staphylococcus epidermidis*	Isolated strain (1)	N	N
68	*Streptococcus salivarius*	Isolated strain (1)	N	N

^a^ATCC, American Type Culture Collection; SG, Shougang Hospital; AB, *Acinetobacter baumannii*; ^b^P, positive; N, negative. Only these strains belonging to the *A. baumannii* could be detected by the MCDA-LFB technique, indicating the extremely high selectivity of the method. Particularly, *pgaD* and *bla*_*OXA-23-like*_ genes can be simultaneously detected and correctly identified in a reaction vessel.

### The clinical application of the duplex-MCDA-LFB assay

In order to evaluate the clinical applicability of the duplex-MCDA-LFB assay, DNA templates extracted from 110 sputum samples were applied to the duplex-MCDA-LFB assay. As show in Table 2, a total of 28 samples were positive for *A. baumannii* strains, in which 26 samples were positive for *bla*_*OXA-23-like*_genes. By the culture-method, 28 strains of *A. baumannii* were isolated from the 110 sputum samples, and 26 strains of *A. baumannii* possessed the *bla*_*OXA-23-like*_gene (Table 2). The 28 sputum samples were positive for *A. baumannii* by culture method, which was consistent with duplex MCDA-LFB detection.

**Table 2:** Clinical evaluation of the MCDA-LFB assay.

Methods	Sputum samples (n = 110)	
	*pgaD* ( + )	*bla*_*OXA-23-like*_ ( + )
Culture	28	26
MCDA	28	26

## Discussion

The widely acquirement of lactamase production capability have jeopardized the treatment effect of most β-Lactams antibiotics, but few of the lactamase can hydrolyze the carbapenem molecules^16^. Thus, carbapenem antibiotics rank the last line antibiotics for the multi-drug resistant treatment, especially for the nosocomial infections in ICU^6,16^. However, resistance to the carbapenem antibioitcs among ESKAPE strains has become an increasingly concern in the past one decade^16–18^. Carbapenem resistant genes were acquired through horizontal transmission or mutation, which encodes carbapenemase and has the ability to hydrolyze the penicillin, cephalosporins and monobactams^16–18^. Among the ESKAPE strains, the carbapenem resistance is much more common among *A. baumannii* strains, the carbapenem resistant genes of which had been found to be able to transmit among different species^19,20^. Efficient identification of the carbapenem-resistant strains have been underlined to be one important strategy for the control of their dissemination and infection^21,22^.*Bla*_*OXA-23-like*_ gene was one important carbapenem resistant gene, and many outbreaks were found to be caused by the *bla*_*OXA-23-like*_-harboring strains^23,24^. Thus, the prompt identification of *A. baumannii* from various samples and detection of carbapenem resistance is essential in cases of suspected infections.

**Figure 6:**
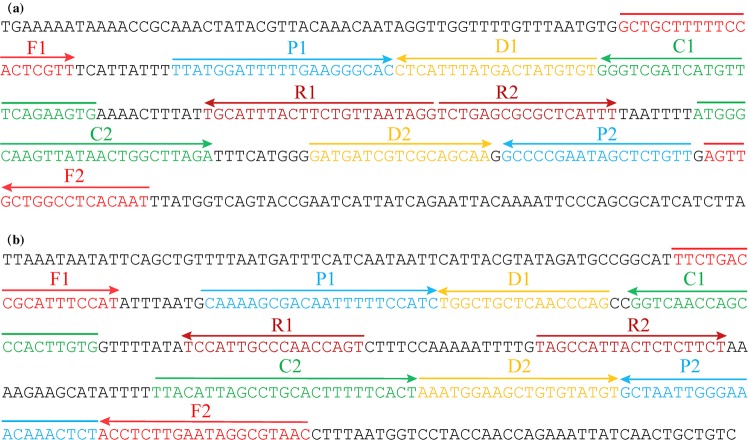
Nucleotide sequence and location of *pgaD* and *bla*_*OXA-23-like*_ genes used to design MCDA-LFB primers. The nucleotide sequences of the sense strands of *pgaD* (**a**) and *bla*_*OXA-23-like*_ (**b**) are showed. The sites of primer sequences were underlined. Left arrows and right arrows showed complementary and sense sequences that are used.

Traditional detection methods, including culture-based techniques, colony morphology, micro-dilution resistance tests, and PCR-based assays (conventional PCR assays and real time PCR methods), are laborious and time-consuming. Herein, a rapid, simple, and specific technique is need for application in a hospital clinical laboratory and a basic laboratory. To obtain more such effective diagnostic tool, we developed a duplex MCDA-LFB method for the detection of *A. baumannii* specific gene *pgaD* and the carbapenem resistant gene *bla*_*OXA-23-like*_. In the MCDA-based system, the primer set of MCDA assay, which specially recognizes ten regions on *pgaD* and *bla*_*OXA-23-like*_ genes, can offer a high degree of specificity (Figs. 5 and 6). The *pgaD*-MCDA primer set were designed based on the species-specific gene *pgaD*, which is unique to all *A. baumannii* isolates. Particularly, carbapenem-resistance in *A. baumannii* is primarily mediated by carbapenemase, which is encoded by *bla*_*OXA-23-like*_ gene. Thus, *bla*_*OXA-23-like*_-MCDA primer set is designed using *bla*_*OXA-23-like*_ gene, which is associated with carbapenem-resistance of *A. baumannii* strains. Analysis of MCDA assay was evaluated with the genomic templates extracted from 52 *A. baumannii* isolates and 15 non-*A. baumannii* strains, and the positive results were obtained from the assay of all *A. baumannii* strains but not for non-*A. baumannii* isolates. The duplex MCDA-LFB method targeting the *pgaD* gene identified *A. baumannii* with 100% specificity, and duplex-MCDA assay targeting the *bla*_*OXA-23-like*_ gene associated with carbapenem resistance identified carbapenem-resistant *A. baumannii* with 100% specificity (Fig. 5). Importantly, the duplex MCDA-LFB assay developed here could detect all *A. baumannii* stains, and identify these strains with the carbapenem resistant gene *bla*_*OXA-23-like*_.

In particular, the lateral flow biosensor (LFB) was employed to analyze the single and duplex MCDA products, because of its rapid results, simple operation, and ease of use in field setting and clinical laboratory. Detection of MCDA product using LFB is not only simpler and less error-prone, but also faster than detection using other analysis methods (such as real time turbidity and colorimetric indicator) employed in the current report. Moreover, lateral flow biosensor is more suitable than other monitoring techniques for simple, rapid and sensitive analysis of single and duplex MCDA products, because LFB eliminates the use of special reagent, instrument and process. LFB used in this study can simultaneously detect and correctly identify two targets in a single examination.

The results readout of the MCDA amplification using the LFBs are mainly based on two important steps: the capture of the amplification products on the test lines and its colorimetric indication^25^. The former step is based on the combination of the antibodies (anti-fluorescein or anti-digoxigenin antibodies embedded onto the test lines of LFBs) and antigens (FITC or digoxigenin labeled onto the amplification products, which will reach the test lines with the syphon reaction)^25^. Thus, the amplified MCDA products of *pgaD* (labeled with FITC and biotin) and *bla*_*OXA-23-like*_ (labeled with digoxigenin and biotin) genes, can be specifically recognized and immobilized onto the TL1 and TL2 of LFBs, respectively. The gold nanoparticle being dyed with the crimson red will show red color when a high quantity of them were gathered together^14^. The high affinity of biotin (labeled onto the amplification products) and streptavidin (coated onto the gold nanoparticles) enable the gold-nanoparticles combined to the amplification products and show red bands on the test lines^26^. Biotins were usually labeled to the 5′-end of the primer, whereas there were occasions that the primer dimer or off-target effect may result in the false positivity of the LFBs^14^. Here we used the single-labeling method with biotins being labeled onto the biotin-14-dUTP in the reaction buffer, which removed the false positivity caused by the hybridization of labeled primers.

Analytical sensitivity of MCDA assays for independently identify *pgaD* and *bla*_*OXA-23-like*_ genes were 100 fg of genomic templates per vessel, and LoD of LFB analysis for MCDA amplicons was in complete accordance with gel electrophoresis detection, colorimetric indicator (MG) analysis and real-time turbidity determination (Fig. 3). Analytical sensitivity of duplex MCDA method for simultaneously detecting *pgaD* and *bla*_*OXA-23-like*_ genes were also 100 fg of DNA templates per reaction, which is conformity with the single *pgaD*-MCDA and *bla*_*OXA-23-like*_-MCDA detection (Figs. 3 and 4). The detection limit of the MCDA primers targeting the *pgaD* gene of *A. baumannii* is more sensitive than the LAMP-based assay for the rapid detection of *A. baumannii* targeting the *bla*_*OXA-51-like*_gene (50 pg per reaction)^27^. Besides, increasing number of studies reported that the *bla*_*OXA-51-like*_ gene is harbored by other species of *Acinetobacter spp*., emphasizing that detection of the *bla*_*OXA-51-like*_ gene on its own is not reliable for the identification of *A. baumannii*^28^. In this study, except for detecting the *pgaD* gene of *A. baumannii*, we developed the duplex MCDA detection for the prevalent carbapenem resistant gene *bla*_*OXA-23-like*_. These results indicate that the duplex-MCDA-LFB method can be good candidate for the rapid detection of *A. baumannii* and the strains harboring the *bla*_*OXA-23-like*_ genes. The feasibility of duplex MCDA-LFB assay was successfully determined using clinical samples, and the results obtained from duplex MCDA-LFB assay are consistent with culture-based methods.

In sum, a duplex MCDA-LFB assay for simultaneous identification of *A. baumannii* strains and carbapenem-resistance based on *pagD* gene and *bla*_*OXA-23-like*_ gene was successfully established. The duplex MCDA-LFB method established in this study displays high selectivity for target gene detection, and the LoD of the method is 100 fg per reaction with pure culture. Lateral flow biosensor is employed for analyzing the MCDA products, which was disposable, easy-to-use and objective. Hence, the duplex MCDA-LFB assay established here was a simple, rapid, sensitive and reliable method to detect all *A. baumannii* strains and identify carbapenem-resistance *A. baumannii* infection for appropriate antibiotic therapy.

## Materials and Methods

### Ethics

All subjects gave their informed consent for inclusion before they participated in the study. The study was conducted in accordance with the Declaration of Helsinki, and the protocol was approved by the Ethics Committee of Peking University Shougang Hospital.

### Reagents and equipment preparation

The main reagents used in this study include the reaction buffer and *Bst* enzyme (Isothermal Amplification Kit, Haitaizhengyuan, Beijing), the Biotin-11-dUTP (Thermo Scientific, Shanghai), the colorimetric indicators (Haitaizhengyuan, Beijing), the nanoparticle LFB, the LFB running buffer, the Nano drop ND-1000 (Calibre, Beijing, China) and a Loopamp Realtime Turbidimeter LA-320C (Eiken Chemical Co., Ltd., Japan.

The LFB was constructed according to the instructions by Wang *et al*.’s report^14^. Briefly, the streptavidin-coated nanoparticles were adhered onto the conjugate pad. On the nitrocellulose membrane pad, there were three lines, including two test lines (conjugated with rabbit anti-fluorescein antibody and sheep anti-digoxigenin antibody, respectively) and one control line (conjugated with biotinylated bovine serum albumin). Finally, the assembled sample pad, conjugate pad, nitrocellulose membrane and the absorbent pad were cut (4 mm in width) and packaged in the plastic shells. The packaged biosensors were stored in dry environment at room temperature. The running buffer was the phosphate buffered saline (PBS) with the pH of 7.4.

### Primers design and screening

Primer Primer 5.0 and PrimerExploer V4 were used for the design of MCDA primers targeting the *A. baumannii* specific gene *pgaD* (Accession No. FJ866500.1) and the carbapenem-resistant gene *bla*_*OXA-23-like*_ (Accession No. NC_025109.1). The designed primers were then blasted on the NCBI to confirm their specificity. The oligomers were synthesized and purified by the company Ruiboxingke (Beijing, China).

Two sets of primers were designed for the two genes, respectively. A clinical-source strain of *A. baumannii* (SG-AB001), which harbors the *bla*_*OXA-23-like*_ gene by the normal polymerase chain reaction (PCR) detection, was used for the primers screening both for the *pgaD* gene and the *bla*_*OXA-23-like*_ gene. The location, the direction, the sequences and the modification of optimal primers were indicated in Fig. 6 and Table 3. Moreover, the genomic templates of *Klebsiella pneumoniae* (*K. pneumoniae*, ATCC2146) and *Staphylococcus aureus* (*S. aureus*) was used as the negative control, with distilled water as the blank control.

**Table 3:** Primers used in the report.

Gene	Primers^a^	Sequences and modifications (5′-3′)^b^	Length^c^
*pgaD*	p-F1	GCTGCTTTTTCCACTCGTT	19 nt
	p-CP1	CACTTCTGAAACATGATCGACCCTTATGGATTTTTGAAGGGCAC	44 mer
	p-C1	CACTTCTGAAACATGATCGACCC	23 nt
	p-C1*	FITC-CACTTCTGAAACATGATCGACCC	28 nt
	p-D1	ACACATAGTCATAAATGAG	19 nt
	p-R1	CCTATTAACAGAAGTAAATGCA	22 nt
	p-R2	TCTGAGCGCGCTCATTT	17 nt
	p-D2	GATGATCGTCGCAGCAA	17 nt
	p-C2	ATGGGCAAGTTATAACTGGCTTAGA	25 nt
	p-CP2	ATGGGCAAGTTATAACTGGCTTAGAAACAGAGCTATTCGGGGC	43 mer
	p-F2	ATTGTGAGGCCAGCAACT	18 nt
*bla*_*OXA-23-like*_	b-F1	TTCTGACCGCATTTCCAT	18 nt
	b-CP1	CACAAGTGGGCTGGTTGACCCAAAAGCGACAATTTTTCCATC	42 mer
	b-C1	CACAAGTGGGCTGGTTGACC	20 nt
	b-C1*	Dig-CACAAGTGGGCTGGTTGACC	24 nt
	b-D1	CTGGGTTGAGCAGCCA	16 nt
	b-R1	ACTGGTTGGGCAATGGA	17 nt
	b-R2	TAGCCATTACTCTCTTCT	18 nt
	b-D2	AAATGGAAGCTGTGTATGT	19 nt
	b-C2	TTACATTAGCCTGCACTTTTTCACT	25 nt
	b-CP2	TTACATTAGCCTGCACTTTTTCACTAGAGTTTGTTTCCCAATTAGC	46 mer
	b-F2	GTTACGCCTATTCAAGAGGT	20 nt

^a^p, *pgaD* gene; b, *bla*_*oxA-23-like*_ gene; p-C1*, 5′-labeled with FITC when used in MCDA-LFB assay; b-C1*, 5′-labeled with Dig when used in MCDA-LFB assay;

^b^FITC, fluorescein isothiocyanate; Dig, digoxigenin;

^c^mer, monomeric unit; nt, nucleotide.

### The reaction systems for the MCDA-LFB assays targeting the *pgaD* and *bla*_*OXA-23-like*_ Genes

In order to examine the feasibility of two sets of MCDA primers, the single MCDA assay either for the *pgaD* gene and the *bla*_*OXA-23-like*_ gene was conducted as the following description. Briefly, the 25 μL reaction system includes 1.6 μM each of CP1 and CP2, 0.4 μM each of F1 and F2, 0.8 μM each of C1*, C2, D1, D2, R1 and R2, 12.5 μL 2 × Reaction Buffer,0.4 mM of biotin-11-dUTP,1.25 μL *Bst* DNA polymerase (10 U) and 1 μL colorimetric indicator (Malachite Green, MG). The amplification mixture of standard MCDA was carried out at 63 °C and then at 85 °C for 5 min to stop the reaction.

For the duplex MCDA-LFB detection of the *pgaD* and the *bla*_*OXA-23-like*_ genes, the 25 μL reaction system included 12.5 μL 2 × Reaction Buffer, 1.25 μL *Bst* DNA polymerase (10 U), 0.8 μM each of p-CP1 and p-CP2, 0.2 μM each of p-F1 and p-F2, 0.4 μM each of p-C1*, p-C2, p-D1, p-D2, p-R1 and p-R2, 1.6 μM each of b-CP1 and b-CP2, 0.2 μM each of b-F1 and b-F2, 0.8 μM each of b-C1*, b-C2, b-D1, b-D2, b-R1 and b-R2,0.4 mM of biotin-11-dUTP and 1 μl DNA template. The duplex MCDA mixtures were carried out at 63^◦^C, and amplification mixtures without the DNA template were used as the blank control. The lowest detectable template amount was tested in triplicate.

A total of four monitoring methods, including real-time turbidity (LA-C320), gel electrophoresis, colorimetric indicator (MG) and lateral flow biosensor (LFB) detection, were used for analyzing the single and duplex MCDA amplicons. During the isothermal amplification, turbidimeter was used to record the real time turbidity changes, which was higher than 0.1 for the positive tubes. By the colorimetric indicator, the positive reactions showed lake green, while the negative reactions faded into colorless. By gel electrophoresis, 3 μL of the MCDA amplification products were embedded into 2% agarose gel and run at 80 volt for 50 minutes. By the LFB, 0.5 μL of the MCDA products were deposited onto the sample pad of the LFB, followed by adding 80 μL of the running buffer (PBS), waiting for 2 to 5 minutes. During the siphon reaction on the LFB, the double strand DNA amplicons of *pgaD* gene, being labeled with FITC and biotin-SA- nanoparticles, will be caught by the first test line (TL 1) which then turns into red^14^. The double strand DNA amplicons of *bla*_*OXA-23-like*_, being labeled with digoxin and biotin-SA-DNPs, will be caught by the second test line (TL 2) and showing the red band. The third line referred to the control line, which will capture the streptavidin-coated nanoparticles, ensuring that the LFB is operating well.

### Screening of optimal amplification temperature

In this report, we explored the optimal amplification temperature for *pgaD*-MCDA and *bla*_*OXA-23-like*_-MCDA primer sets. MCDA amplifications were performed at a constant temperature from 61 °C to 65 °C for 60 min, then incubated at 85 °C for 5 min to stop the MCDA reaction. MCDA mixtures with 1 μL double distilled water (DW) were used as blank control (BC), and 1 μL of DNA templates derived from *K. pneumoniae* and *S. aureus* were selected as negative controls (NC).

### Sensitivity of the primers for MCDA test

DNA templates of strain SG-AB001, which harbor both the *pgaD* and *bla*_*OXA-23-like*_ genes, was serially diluted with distilled water ranging from 10 ng to 1 fg. The single and duplex MCDA-LFB assays were conducted by adding 1 μL of the diluted DNA into the reaction systems. Analytical sensitivity evaluation was repeated for three times.

### Specificity of the duplex MCDA assay

DNA templates extracted from 53 strains of *A. baumannii*, which were isolated from clinical patients, were firstly tested for the presence of *bla*_*OXA-23-like*_ with PCR screening. The PCR tests were performed according to *Li, et al*.’s reports^27^. Then, these strains were applied for the duplex-MCDA-LFB test for *pgaD* and *bla*_*OXA-23-like*_ genes. Moreover, another 15 strains of non-*A. baumannii* were tested for the duplex MCDA-LFB to verify the specificity of the primers for the *pgaD* and *bla*_*OXA-23-like*_ genes. All the tests were repeated in triplicate.

### The clinical application of the duplex-MCDA-LFB assay

In order to validate the clinical feasibility of the duplex MCDA technique developed in this report, DNA templates of 110 clinical sputum samples were used to evaluate the duplex MCDA-LFB assay. Particularly, these clinical sputum samples have been cultured for *A. baumannii* with the traditional clinical cultural-based methods. The results of the duplex MCDA-LFB were further confirmed with the single MCDA-LFB tests for the *pgaD* gene and *bla*_*OXA-23-like*_ gene, independently. All the genomic-based tests were repeated twice independently.

## Supplementary information

Supplementary Information file

## Data Availability

The datasets used during the current study are available from the corresponding author on reasonable request.
