# Supplementary material for: Identification of Acinetobacter baumannii and its carbapenem-resistant gene blaOXA-23-like by multiple cross displacement amplification combined with lateral flow biosensor
Source: Sci Rep. 2019 Nov 29;9:17888. doi: 10.1038/s41598-019-54465-8 (PMC6884502; doi:10.1038/s41598-019-54465-8)
Supplement: Supplementary file 1 — Supplementary Information file [file 41598_2019_54465_MOESM1_ESM.pdf]

Identification of *Acinetobacter baumannii* and its carbapenem-resistant gene *bla*$_{OXA\text{-}23\text{-}like}$ by multiple cross displacement amplification combined with lateral flow biosensor

Authors:

Shoukui Hu[1,†], Lina Niu[1,2,†], Fan Zhao[1,†], Linlin Yan [1,†], Jinqing Nong [1], Chunmei Wang[1], Naishu Gao [1], Xiaoxue Zhu [1], Lei Wu [1], Tianhui Bo [1], Hongyu Wang [1], Jin Gu [1]*

(a)

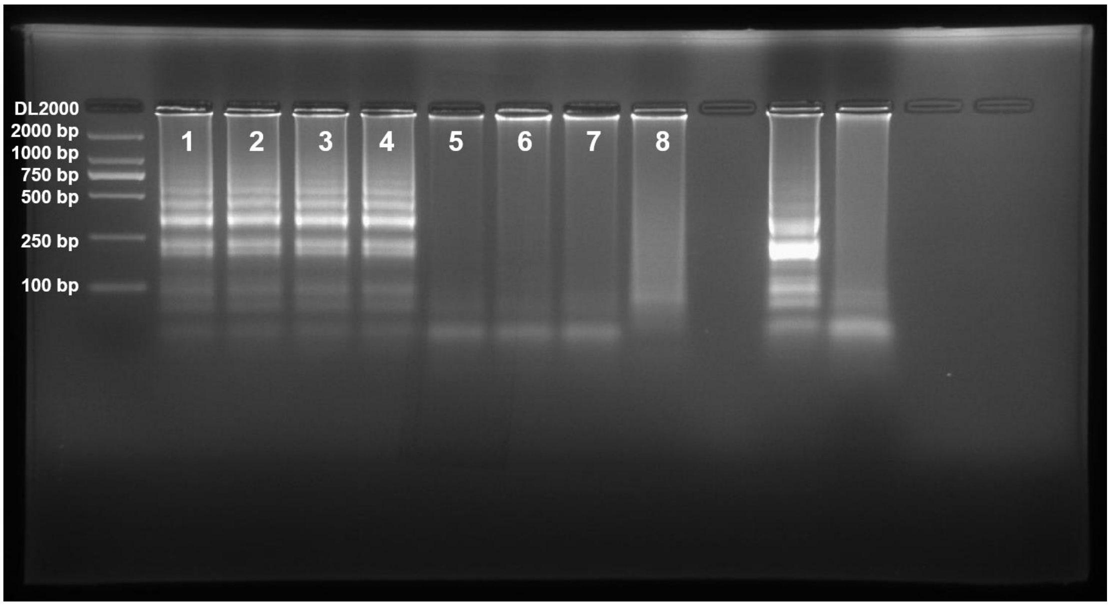

(b)

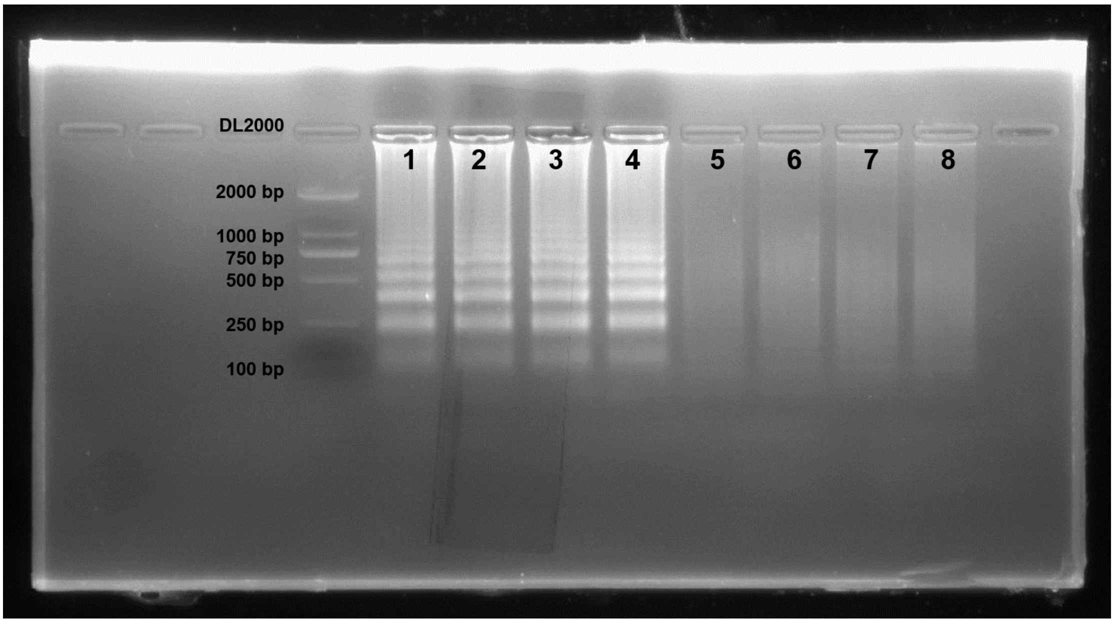

Figure S1. Detection of a single target in a MCDA reaction. Two sets of MCDA

primers targeting the *pgaD* (a) and *bla*$_{OXA\text{-}23\text{-}like}$ (b) genes were used in different reactions and the serial dilutions (10 ng, 10 pg, 1 pg, 100 fg, 10 fg and 1 fg) of target templates were subjected to MCDA reactions. (a) and (b), gel electrophoresis applied for analysis of *pgaD*- and *bla*$_{OXA\text{-}23\text{-}like}$-MCDA products. Signals/Tubes/Biosensors/Lanes 1-6: *A. baumannii* (SG-AB001) genomic templates (10 ng-1fg); Signal/Tube/Biosensor/Lane 7: negative control (*K. pneumoniae*); Signal/Tube/Biosensor/Lane 8: blank control (DW).
